# Shared alterations in resting-state brain connectivity in adults with attention-deficit/hyperactivity disorder and their unaffected first-degree relatives

**DOI:** 10.1017/S0033291719003374

**Published:** 2021-01

**Authors:** Valentino Antonio Pironti, Deniz Vatansever, Barbara Jacquelyn Sahakian

**Affiliations:** 1 Department of Psychiatry, School of Clinical Medicine, University of Cambridge, Cambridge, UK; 2 Behavioural and Clinical Neuroscience Institute, University of Cambridge, Cambridge, UK; 3 Suno Innova Ltd, Unit 6, 109 Cambridge Road Industrial Estate, Cambridge, UK; 4 Institute of Science and Technology for Brain-inspired Intelligence, Fudan University, Shanghai, PR China; 5 Division of Anaesthesia, School of Clinical Medicine, University of Cambridge, Cambridge, UK; 6 Department of Psychology, University of York, Heslington, York, UK

**Keywords:** Attention-deficit/hyperactivity disorder, default mode network, dimensional, endophenotype, functional connectivity, resting state, sustained attention

## Abstract

**Background:**

Attention-deficit/hyperactivity disorder (ADHD) is a developmental condition that often persists into adulthood with extensive negative consequences on quality of life. Despite emerging evidence indicating the genetic basis of ADHD, investigations into the familial expression of latent neurocognitive traits remain limited.

**Methods:**

In a group of adult ADHD probands (*n* = 20), their unaffected first-degree relatives (*n* = 20) and typically developing control participants (*n* = 20), we assessed endophenotypic alterations in the default mode network (DMN) connectivity during resting-state functional magnetic resonance imaging in relation to cognitive performance and clinical symptoms. In an external validation step, we also examined the dimensional nature of this neurocognitive trait in a sample of unrelated healthy young adults (*n* = 100) from the Human Connectome Project (HCP).

**Results:**

The results illustrated reduced anti-correlations between the posterior cingulate cortex/precuneus and right middle frontal gyrus that was shared between adult ADHD probands and their first-degree relatives, but not with healthy controls. The observed connectivity alterations were linked to higher ADHD symptoms that was mediated by performance in a sustained attention task. Moreover, this brain-based neurocognitive trait dimensionally explained ADHD symptom variability in the HCP sample.

**Conclusions:**

Alterations in the default mode connectivity may represent a dimensional endophenotype of ADHD, hence a significant aspect of the neuropathophysiology of this disorder. As such, brain network organisation can potentially be employed as an important neurocognitive trait to enhance statistical power of genetic studies in ADHD and as a surrogate efficacy endpoint in the development of novel pharmaceuticals.

## Introduction

Attention-deficit/hyperactivity disorder (ADHD) is a childhood onset neurodevelopmental condition, characterised by clinical symptoms of impulsivity, hyperactivity and inattention as well as emotional dysregulation (Kieling et al., 2010) that often persists into adulthood (Faraone, 2007) with far-reaching negative consequences on quality of life (Barkley & Fischer, 2010). In addition to the clinical symptoms and deficits in core cognitive functions (McLean et al., 2004), emerging research now indicates anatomical and neurophysiological abnormalities associated with this disorder. Reductions in grey matter volume and cortical thickness (Carmona et al., 2005; Shaw et al., 2006, 2007), disruptions to white matter tracts (Valera, Faraone, Murray, & Seidman, 2007) and aberrant brain functional network organisation (Konrad & Eickhoff, 2010) have all been suggested to form the neuropathophysiology of ADHD. Collectively, this evidence indicates wide-spread dysfunctions in core neuronal circuits, highlighting the biological underpinnings of ADHD symptomatology.

In this regard, a recent review on the genetic basis of ADHD indicates the mean heritability across 37 twin studies as 74% (Faraone & Larsson, 2019). Furthermore, in a population-based family sample, greater genetic relatedness was linked to greater familial aggregation of ADHD with a heritability estimate of around 80% that was heightened with the persistence of ADHD into adulthood (Chen et al., 2017). However, despite this reported heritability (Franke et al., 2012; Mick & Faraone, 2008; Thapar, O'Donovan, & Owen, 2005), genetic studies for the identification of ADHD susceptibility genes have so far produced disparate results (Hinney et al., 2011). This might possibly be due to the heterogeneity of the disorder, intrinsic limitations of the commonly employed nosographic systems, experimental focus that is largely confined to young children, and limited reports on endophenotype-based research, which requires further investigation.

Defined as covert, quantitative traits, endophenotypes are assumed to be highly heritable and closely related to the expression of genes linked to a disorder (Cannon & Keller, 2006). Furthermore, the number of genes associated with such basic neurocognitive components are expected to be fewer as compared with those linked to the overt clinical phenotype, which enhances the statistical power in studies aimed at discovering susceptibility genes (Gottesman & Gould, 2003). Thus, similar to the strategies employed in assessing ‘at-risk’ individuals in other mental health disorders such as schizophrenia (Whitfield-Gabrieli et al., 2009), further research into familial endophenotypes that incorporate parents, full siblings or children who share 50% of their genetic makeup with the patients, may provide a more fruitful route to identifying genes that will ultimately improve translational research and drug discovery (Castellanos & Tannock, 2002). This better understanding of the neuropathophysiology of ADHD should in turn improve success rates in the search for novel pharmaceutical agents (Doyle et al., 2005).

To this end, emerging familial investigations have recently illustrated shared abnormalities in sustained attention performance (Gau & Huang, 2014) and brain morphology (Pironti et al., 2014) between ADHD patients and their first-degree relatives that may potentially make up vital endophenotypes of ADHD. In line with these findings, another latent neurocognitive trait may be rooted in the intrinsic brain connectivity dynamics as measured via resting-state functional magnetic resonance imaging (rs-fMRI). Termed functional connectivity (Friston, 1994), investigations into the temporal correlations of spontaneous blood-oxygen-level-dependent (BOLD) signal fluctuations from distinct brain regions (Biswal, Yetkin, Haughton, & Hyde, 1995) have been largely popularised for their utility in identifying modifications to the intrinsic brain functional network organisation across mental health disorders (Fox & Greicius, 2010). Specifically, one particular large-scale system, namely the default mode network (DMN), has been recognised as a new locus of dysfunction in ADHD, with studies reporting on its altered levels of integrity and connectivity during wakeful states of rest (Castellanos, Kelly, & Milham, 2009; Konrad & Eickhoff, 2010; Posner, Park, & Wang, 2014).

Comprising the anterior/posterior midline as well as the posterior parietal cortices, the DMN has been associated with internal, self-referential thoughts during unconstrained resting-state conditions (Andrews-Hanna, Smallwood, & Spreng, 2014) with further implications in executive functions commonly impaired in ADHD such as sustained attention, working memory and cognitive flexibility (Bozhilova, Michelini, Kuntsi, & Asherson, 2018; McLean et al., 2004). Across both adults (Castellanos et al., 2008) and adolescents (Sun et al., 2012) with ADHD, previous research has shown significant reductions in anti-correlations (or negative connectivity) of a core DMN hub, namely the posterior cingulate cortex and precuneus (PCC/PCUN) (Fransson & Marrelec, 2008), and regions commonly implicated in cognitive control. In combination with these reports, the significant heritability estimate of DMN in the general population (Glahn et al., 2010) suggests the potentially utility of this large-scale network as putative brain-based endophenotype under neurodevelopmental conditions (Korgaonkar, Ram, Williams, Gatt, & Grieve, 2014). Nevertheless, endophenotype-based research studies into the neuropathophysiology of this network across ADHD probands and their first-degree relatives in comparison with typically developing controls, as well as direct investigations into its cognitive and clinical correlates, have not been previously conducted.

To this end, here we investigated alterations in brain functional network connectivity within the DMN during rs-fMRI and tested the suitability of this measure as a potential neurocognitive endophenotype in a cohort of 20 adult ADHD probands, their 20 unaffected first-degree relatives and 20 typically developing control participants (*n* = 60). Given prior research, we hypothesised that alterations in the connectivity of DMN to the frontal brain regions would be shared between adults with ADHD and their first-degree relatives, which would significantly correlate with deficits in sustained attention performance and severity of ADHD symptoms. Finally, based on recent reports that highlight ADHD symptomatology as dimensional traits across a continuum of normal to abnormal functioning (Chabernaud et al., 2012; Elton, Alcauter, & Gao, 2014), in a separate dataset from the Human Connectome Project (HCP) (*n* = 100) we aimed to assess the generalisability of our findings by examining the potential utility of this neurocognitive trait in explaining dimensional variation in ADHD symptomatology in a cohort of unrelated healthy young adults.

## Methods

### Participants and clinical assessment

Ethical approval for this experiment was obtained from the Cambridgeshire 3 Research Ethics Committee (REC: 09/H0306/38). All individuals gave informed consent prior to their participation. ADHD probands and their first-degree relatives were recruited from the Adult ADHD Research Clinic, Addenbrooke's Hospital, Department of Psychiatry, University of Cambridge, whereas typically developing controls were recruited from the local community.

For the patient cohort, the diagnosis of ADHD (DSM-IV Text Revision) was based on a full clinical interview with the patient and an informant who had known the patient since childhood (e.g. parents). The clinical assessment included Barkley Adult ADHD Rating Scale Version IV (BAARS-IV) for childhood and adult symptoms, provided by both the patient and the informant (Barkley, 2011). In addition, the Mini International Neuropsychiatric Interview (MINI) (Sheehan et al., 1998) was administered to screen for DSM-IV Axis I disorders, and the National Adult Reading Test (NART) (Bright, Jaldow, & Kopelman, 2002) was used to estimate full scale IQ. Eligible ADHD patients were then asked to contact a first-degree relative (e.g. parents, full siblings). Both the first-degree relatives of the ADHD probands and the typically developing control participants were screened for undiagnosed adult ADHD or a comorbid disorder using the same clinical assessments, except for only the self-report BAARS-IV (Barkley & Murphy, 2006) for adult symptoms was administered in these groups, without the informant.

The exclusion criteria were: NART full scale IQ score equal to or less than 90, current or past history of developmental, neurological or psychiatric disorders based on a formal DSM-IV Text Revisions diagnosis and contraindication to an MRI scan. The final group of ADHD participants recruited for this study was 20 (mean age = 32.20 years, s.d. = 10.31, 3/17 female to male ratio), comprising 16 patients with combined type and four with inattentive type; 16 of which were medicated with methylphenidate, while four were not receiving any medication for their ADHD diagnosis. In addition to the patient group, a total of 20 age and gender-matched first-degree relatives (mean age = 38.85 years, s.d. = 15.31, 10/10 female to male ratio) of the ADHD probands and 20 typically developing control participants (mean age = 32.5 years, s.d. = 5.8, 7/13 female to male ratio) were recruited for this study. Neither the first-degree relatives, nor the typically developing control participants showed ADHD symptoms meeting the DSM-IV Text Revision diagnostic threshold for ADHD, or a diagnosis of any other comorbid disorder. With the aim of minimising the potential impact of psychotropic medication on cognitive performance and brain dynamics, ADHD participants were asked to withhold taking their medication 24 h before (Gualtieri et al., 1982; Turner, Blackwell, Dowson, McLean, & Sahakian, 2005) and to refrain from consuming alcohol or caffeine-containing drinks on the day of the testing. The same request was made for the first-degree relatives and control participants.

In order to ensure matching of demographic information between groups, age, gender and NART full scale IQ scores were entered into multivariate and univariate analyses of variance (ANOVA) and post-hoc *t* tests (online Supplementary Table S1) using the SPSS Statistics software package (Version 23.0) (https://www.ibm.com/products/spss-statistics). The self-report BAARS-IV current total symptom scores as well as the inattentive and hyper-impulsive sub-scales were also assessed for group differences with the same statistical procedures, correcting for age, gender and NART full scale IQ scores (online Supplementary Table S2).

### Cognitive assessment

All participants completed the Rapid Visual Information Processing (RVP) test of sustained attention from the Cambridge Neuropsychological Test Automated Battery (CANTAB) (Robbins, James, Owen, & Sahakian, 1994; Sahakian & Owen, 1992). In this task, participants were asked to pay attention to a computer screen in which pseudo-random digits [2–9] were presented one at a time, at a frequency of 100 per min, next to a list of three-digit sequences. Participants had to press a button any time they detected one of the three-digit target sequences. Requiring both sustained attention and working memory, performance in this task has been previously shown to be lower in ADHD patients and their siblings as compared to typically developing controls (Gau & Huang, 2014). Univariate analysis of covariance (ANCOVA) was used to assess group differences in performance, with group membership (i.e. ADHD probands, their first-degree relatives, healthy controls) entered as fixed factor and total hits in the RVP task as the dependent variable. Age, gender and NART full scale IQ scores were included as covariates and post-hoc *t* tests were corrected for multiple comparisons using the Bonferroni method.

### MRI data acquisition and preprocessing

Participants were scanned using a Siemens MAGNETOM Tim Trio 3T scanner (32-channel head coil) at the Wolfson Brain Imaging Centre, Cambridge. The brain imaging session started with a high resolution T1-weighted, magnetisation-prepared rapid gradient-echo (MPRAGE) structural scan (TR = 2300 ms, TE = 2.98 ms, slice thickness = 1.00 mm). The echo planar imaging sequence parameters for the 8.73-min eyes-closed resting-state functional data acquisition were as follows: 32 slices in each volume, 3.0 mm slice thickness, 3.0 × 3.0 × 3.0 voxel size, TR = 2000 ms, TE = 30 ms, flip angle = 78 degrees, 262 volumes. The MRI data preprocessing pipeline included slice-time and motion (six degrees of freedom) corrections, co-registration to the high resolution T1-weighted structural image, normalisation to the Montreal Neurological Institute (MNI) space through the unified segmentation–normalisation algorithm (Ashburner & Friston, 2005) and smoothing with an 8 mm FWHM Gaussian kernel using the SPM software package (Version 12.0) (http://www.fil.ion.ucl.ac.uk/spm/), based on the MATLAB platform (Version 15a) (http://www.mathworks.co.uk/products/matlab/).

### Functional connectivity analysis

Seed-based functional connectivity analyses were shown to be influenced by the definition of seed regions of interest (ROI) (Smith et al., 2011), which can be partially alleviated by the use of large-scale meta-analyses. For this reason, we employed an objective approach to define our seed ROI using Neurosynth (http://neurosynth.org/) (Yarkoni, Poldrack, Nichols, Van Essen, & Wager, 2011). Following the meta-analysis performed on fMRI brain activity data for three search terms stored in the Neurosynth database, namely ‘default (*n* = 519), default mode (*n* = 419), default network (*n* = 80)’, three false-positive discovery rate corrected, statistical co-activation maps (P term|activation) were created. The three maps corresponding to the three search terms were binarised, overlapped and masked with an anatomical automated labelling atlas-based PCC/PCUN template obtained from the Wake Forest University PickAtlas toolbox (http://fmri.wfubmc.edu/software/pickatlas) (Maldjian, Laurienti, Kraft, & Burdette, 2003). This procedure provided us with a PCC/PCUN seed that was previously utilised in a resting-state study (Vatansever, Manktelow, Sahakian, Menon, & Stamatakis, 2016). PCC, together with PCUN (Fransson & Marrelec, 2008) is considered to be one of the most robust DMN hubs (Leech & Sharp, 2014), hence providing a reliable seed region to assess DMN connectivity.

The functional connectivity analysis was carried out using the *Conn* functional connectivity toolbox (Version 17.f) (https://www.nitrc.org/projects/conn) (Whitfield-Gabrieli & Nieto-Castanon, 2012). At first, a strict pipeline of denoising procedure was employed that included CompCor components attributable to the signal from white matter and cerebrospinal fluid (Behzadi, Restom, Liau, & Liu, 2007) as well as a linear detrending term, eliminating the need for global signal normalisation that reportedly enhances spurious anti-correlations (Chai, Castanon, Ongur, & Whitfield-Gabrieli, 2012; Murphy, Birn, Handwerker, Jones, & Bandettini, 2009). The subject-specific six realignment parameters, the main effect of rest-condition and their second-order derivatives were also included in the analysis as potential confounds (Fair et al., 2007). In an additional step to remove potential motion artefacts, the data were motion scrubbed using the composite motion score. The settings of a global signal greater than *z* = 9 and head motion greater than 2 mm were used to identify volumes with high composite motion score which were then added as potential confounds to the linear regression. Furthermore, mean framewise displacement, denoting scan-to-scan motion, was also calculated to be used as a covariate in our subsequent correlation analyses. Moreover, a temporal filter of 0.009 and 0.08 Hz was applied to focus on low frequency fluctuations (Fox et al., 2005).

Following this procedure, seed-based functional connectivity analyses were performed for each subject using the average BOLD signal from the binarised ROI map described above. One-sample *t*-statistics were used to obtain PCC/PCUN connectivity maps representing the DMN and its anti-correlations for each group. All reported clusters were multiple-comparison corrected using the family-wise error detection technique at the 0.05 level of significance (voxel-level 0.001 uncorrected). Moreover, group-level ANOVA was carried out using *F*-statistics to examine changes in DMN connectivity (PCC/PCUN seed) across ADHD probands, their first-degree relatives and the typically developing control group. The individual connectivity values (*z*-scores) extracted from the cluster that showed significant alterations in connectivity to the chosen seed were then entered into a univariate ANCOVA (correcting for age, gender and NART full scale IQ score) to further examine the fixed-effects of group membership on DMN connectivity. Post-hoc *t* tests were carried out to assess pair-wise group differences that were corrected using the Bonferroni method.

### Brain, cognition and symptom correlation analysis

Across the whole study cohort, the association between PCC/PCUN connectivity to the brain regions which showed a significant group interaction (*z*-scores), BAARS-IV current total symptom scores and the number of total hits from the RVP sustained attention task was examined using bivariate non-parametric partial Spearman's correlations (correcting for age, gender, NART full scale IQ score and mean framewise displacement). All measures were assessed for normality using the Shapiro–Wilk test and *Q*–*Q* plots, and the correlations were Bonferroni corrected for multiple-comparisons. The results were visualised using the Seaborn package (Waskom et al., 2018) from Python library. In addition, a mediation analysis was performed with the RVP task (total hits) sustained attention scores as the mediator using PROCESS macro Version 3 (Hayes, 2018) with a percentile bootstrap estimation approach (10 000 samples) (Shrout & Bolger, 2002).

### External validation dataset and analysis

An independent dataset from the HCP was used to provide external validation to our results from the main experiment, to assess generalisability of our findings and to test the potential utility of the identified neurocognitive traits in explaining dimensional variation in ADHD symptomatology across a group of unrelated healthy young adult participants. A total of 100 participants' high-quality, openly available data were downloaded from the HCP Q5 Release (mean age = 29.11 years, s.d. = 3.676, 54/46 female to male ratio). Full details on the HCP dataset, including the acquisition parameters are provided in the literature (Van Essen et al., 2013). The output from the HCP minimal preprocessing pipeline was used for the analyses. Specifically, T1-weighted images from the output of the HCP *PreFreeSurfer* pipeline that was biased corrected and normalised to the MNI space were utilised as high-resolution structural images. Similarly, the functional data output of the *fMRIVolume* pipeline from two-runs of 15-min rs-fMRI sessions that was artefact/motion corrected and normalised to the MNI space was used for the seed-based functional connectivity analyses. This preprocessing pipeline largely matched the one used for the main experimental data besides few exceptions mentioned below.

The high-quality HCP data were acquired with a very short, 0.72 s TR meaning each slice in a given volume was obtained highly close together, thus eliminating the need for slice-time correction (Glasser et al., 2013). In addition, previous connectivity studies using HCP data have shown that spatial smoothing did not alter the results and thus no smoothing was used (Finn et al., 2015). Finally, instead of the typical anterior–posterior or posterior–anterior phase encoding directions, the HCP rs-fMRI images were acquired with left–right (LR) and right–left (RL) phase encoding directions using an asymmetric acquisition matrix with the purpose of reducing signal loss and distortions that result from high-resolution data acquisition. Hence, functional data from both the LR and RL phase-encoding runs were used to calculate average functional connectivity maps (Glasser et al., 2013; Van Essen et al., 2013).

Subsequently the same data denoising procedures (except for scrubbing) and functional connectivity analyses from the main experimental study was employed for the HCP functional data. A one-sample *t* test was performed in order to assess DMN connectivity, and a binarised mask for the region that showed significant alterations in connectivity between groups from the main experiment was used to extract connectivity values (*z*-scores). We then employed bivariate non-parametric partial Spearman's correlation to assess the link between DMN connectivity and Achenbach Adult Self-Report (ASR) DSM-oriented Attention Deficit/Hyperactivity (AD/H) scale (Achenbach & Rescorla, 2003) within the upper 50th percentile of the full cohort, who scored relatively high on ADHD symptoms (corrected for age, gender and mean framewise displacement). This cut-off point was chosen to avoid floor effects in this neurotypical cohort (mean age = 28.98 years, s.d. = 3.583, female to male ratio = 22/28).

Finally, the cluster of brain regions showing significant alteration in its connectivity to the chosen seed from the main experiment was decoded for cognitive terms using Neurosynth meta-analysis toolbox (Rubin et al., 2017). The top 100 terms were chosen, from which we removed anatomical terms such as ‘prefrontal cortex’, leaving behind only the cognitive terminology. These terms were then entered into a word cloud with the associated weights. A total of 23 cognitive terms (after removal of anatomical terms) most associated with activity in this cluster of brain regions was used to create a word cloud to aid in the cognitive interpretation of our results.

## Results

### Default mode connectivity alterations

**Fig. 1.**
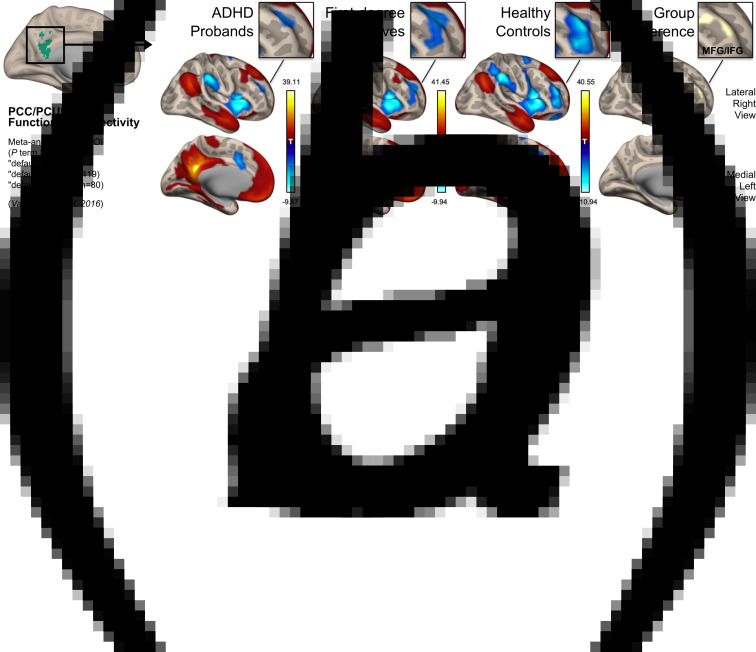
Group differences in resting-state default mode connectivity in ADHD probands, their unaffected first-degree relatives and typically developing control participants. (*a*) An overlap of the meta-analytic activation maps related to the terms ‘default, default mode and default network’ from the Neurosynth database was used to create an objective seed of the PCC/PCUN for seed-based functional connectivity analysis of the DMN. (*b*) Group-level one sample *t* tests and an *F*-contrast revealed consistent yet altered DMN connectivity across the three groups (displayed on an inflated brain surface from the Montreal Neurological Institute – MNI). Specifically, the group difference was centred on the right middle frontal gyrus (MFG) [MNI peak: +48 + 48 + 16, cluster size = 414 voxels], extending into the inferior frontal gyrus (IFG).

**Fig. 2.**
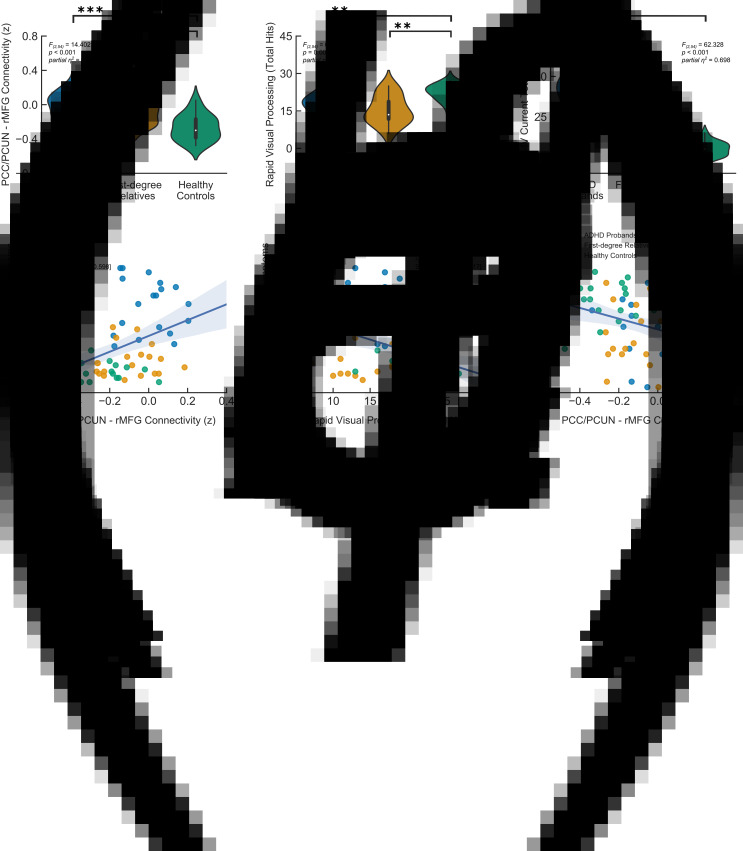
Shared neurocognitive impairments in adults with ADHD and their unaffected first-degree relatives in relation to cognitive performance and ADHD symptomatology. (*a*) Default mode functional connectivity as characterised by PCC/PCUN to right MFG interactions and (*b*) cognitive assessment on the RVP task of sustained attention revealed neurocognitive endophenotypes i.e. traits that are potentially closer to the genetic expression as opposed to the clinical outcome. (*c*) All groups were also assessed for ADHD symptoms using Barkley Adult ADHD Rating Scale Version IV (BAARS-IV) self-reports. In line with the clinical diagnosis, the ADHD probands scored higher than both their first-degree relatives and typically developing control participants, whereas no significant difference was observed between the latter two groups. (*d*–*f*) Non-parametric bivariate partial Spearman's correlations (*r*_s_) across the three experimental groups illustrated significant links between brain connectivity, cognitive performance and ADHD symptom scores (corrected for age, gender, NART full scale IQ scores and mean framewise displacement). In other words, participants with higher BAARS-IV current total symptom scores showed reduced DMN anti-correlation and a smaller number of total hits on the RVP sustained attention task, which in turn was linked to reduced DMN anti-correlation. The violin plots illustrate the kernel density estimation of the underlying distribution, whereas the boxplots show the interquartile range. *** denotes *p* < 0.001 and ** denotes *p* < 0.01. The shaded areas represent 95% CIs.

In line with previous studies, our PCC/PCUN seed-based functional connectivity analysis (Fig. 1*a*) revealed an intact DMN across all three groups, covering regions of the posterior cingulate, medial prefrontal cortices, bilateral angular gyri, anterior/medial temporal lobes and superior frontal gyri (Fig. 1*b*). However, an ANCOVA across the three groups with DMN connectivity as the dependent variable highlighted a cluster of brain regions centred on the right middle frontal gyrus (MFG) [extending into the inferior frontal gyrus (IFG)] [MNI peak: +48 + 48 + 16, cluster size = 414 voxels] as showing significant alterations in its connectivity to the chosen seed ROI (*F*_(2,54)_ = 14.402, *p* < 0.001, *partial η*^2^ = 0.348) (Fig. 1*b*). Post-hoc *t* tests on the connectivity values extracted from this cluster showed a significant difference (i.e. reduced anti-correlation) between ADHD probands and controls [*t*_(54)_ = 5.288, 95% confidence interval (CI) (0.163–0.363), *p* < 0.001, *partial η*^2^ = 0.341] as well as between first-degree relatives and controls [*t*_(54)_ = 3.440, 95% CI (0.071–0.271), *p* = 0.001, *partial η*^2^ = 0.180]; but there were no significant differences between the ADHD probands and their first-degree relatives [*t*_(54)_ = 1.844, 95% CI (−0.008 to 0.192), *p* = 0.071, *partial η*^2^ = 0.059] (Table 1, Fig. 2*a*).

**Table 1.** Statistical comparison of resting-state brain connectivity, cognitive performance and symptom scores across ADHD probands, their unaffected first-degree relatives and typically developing control participants, corrected for age, gender and NART full scale IQ score.

	ADHD probands		First-degree relatives		Healthy controls		ANCOVA		
	Mean	s.d.	Mean	s.d.	Mean	s.d.	*F*	*p*	*partial η*^2^
Brain connectivity PCC/PCUN to rMFG (*z*)	−0.02	0.14	−0.10	0.13	−0.27	0.15	14.40	<0.001	0.348
Cognition RVP (total hits)	15.95	5.22	15.35	5.67	22.0	3.57	6.30	0.003	0.189
ADHD symptomatology BAARS-IV (current total symptoms)	36.15	12.39	10.00	7.38	5.20	4.29	62.33	<0.001	0.698

PCC/PCUN, posterior cingulate cortex/precuneus; rMFG, right middle frontal gyrus; RVP, rapid visual information processing; BAARS-IV, Barkley Adult ADHD Rating Scale Version IV.

### Cognitive and clinical assessments

In parallel to the DMN connectivity results, ANCOVA for the sustained attention scores showed a significant difference in the RVP total hits across the three experimental groups (*F*_(2,54)_ = 6.300, *p* = 0.003, *partial η*^2^ = 0.189), in which both the ADHD probands [*t*_(54)_ = −3.037, 95% CI (−7.477 to −1.531), *p* = 0.004, *partial η*^2^ = 0.146] and their unaffected first-degree relatives [*t*_(54)_ = −3.110, 95% CI (−7.595 to −1.641), *p* = 0.003, *partial η*^2^ = 0.152] performed significantly worse than the typically developing control participants, whereas the former two groups showed no significant difference to each other [*t*_(54)_ = −0.077, 95% CI (−3.088 to 2.861), *p* = 0.939, *partial η*^2^ < 0.001] (Table 1, Fig. 2*b*).

In contrast, an ANCOVA between groups on the BAARS-IV current symptom scores showed a significant group difference (*F*_(2,54)_ = 62.328, *p* < 0.001, *partial η*^2^ = 0.698) that diverged away from brain connectivity and sustained attention task performance results (Table 1, Fig. 2*c*). Post-hoc *t* tests revealed that the ADHD probands scored significantly higher than both their first-degree relatives [*t*_(54)_ = 8.982, 95% CI (21.061–33.165), *p* < 0.001, *partial η*^2^ = 0.599] and typically developing controls [*t*_(54)_ = 10.229, 95% CI (24.816–36.916), *p* < 0.001, *partial η*^2^ = 0.660]; however, the latter two groups did not show a significant difference to each other [*t*_(54)_ = 1.241, 95% CI (−2.304 to 9.811), *p* = 0.220, *partial η*^2^ = 0.028]. Similar results were obtained for the inattentive and hyperactive-impulsive sub-scales (online Supplementary Table S2).

### Association of brain connectivity, cognition and ADHD symptomatology

Across all groups, the BAARS-IV current total symptom scores showed a significant correlation with PCC/PCUN to right MFG connectivity [*partial r*_s_ = 0.405, 95% CI (0.168–0.598), *p* = 0.002] (Fig. 2*d*). Moreover, there was a significant negative correlation between the PCC/PCUN to right MFG connectivity and total hits in the RVP sustained attention task [*partial r*_s_ = −0.360, 95% CI (−0.563 to −0.117), *p* = 0.006] (Fig. 2*e*), which was in turn negatively correlated with BAARS-IV current total symptom scores [*partial r*_s_ = −0.456, 95% CI (−0.636 to −0.229), *p* < 0.001] (Fig. 2*f*, online Supplementary Table S3), corrected for age, gender and mean framewise displacement. Findings from the inattentive and hyperactive-impulsive sub-scales were in line with the main results (online Supplementary Tables S4 and S5). In addition, a mediation analysis indicated that there was a significant indirect effect of PCC/PCUN to right MFG connectivity on BAARS-IV total current symptom scores through the participants' performance (total hits) on the RVP sustained attention task [*b* = 8.054, 95% CI (0.920–19.3727)]. In other words, DMN connectivity was linked to ADHD symptom scores that were approximately 8.1 points higher as mediated by attentional performance.

### Generalisability to an independent dataset

**Fig. 3.**
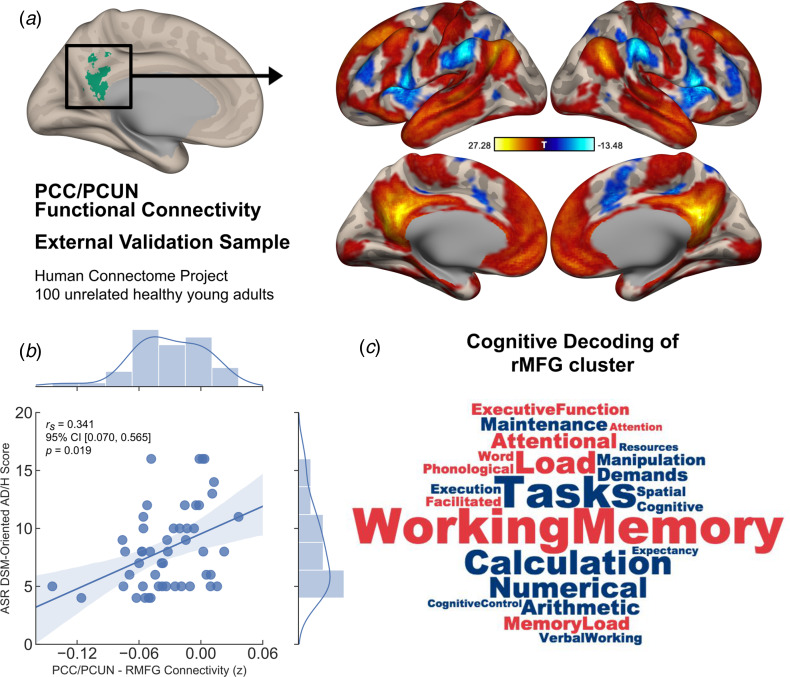
Generalisability of the identified neurocognitive link as a dimensional trait in an independent sample of unrelated healthy young adults. (*a*) Default mode connectivity as revealed by PCC/PCUN seed-based functional interactions across two runs of 15-min resting-state sessions in a sample of 100 unrelated participants from the HCP showed a similar pattern of connectivity as identified in the main experimental study. (*b*) In the upper 50th percentile of high symptom scorers, Achenbach Adult Rating Scale (ASR) DSM-oriented AD/H scores and the PCC/PCUN anti-correlation with the right MFG cluster showed a significant link, in which reduced anti-correlation was associated with greater AD/H symptoms (*r*_s_ = partial Spearman's correlation corrected for age, gender and mean framewise displacement). (*c*) Cognitive terms that were most commonly associated with activation in the right MFG was extracted from the Neurosynth database. This right hemispheric cluster depicted strong association with working memory, attentional load, task demands and other higher executive functions. While the font size of the terms indicates strength, the colour represents directionality of the loadings (red = positive, blue = negative). The shaded areas represent 95% CIs.

Similar to the results obtained from our main experimental study, the PCC/PCUN seed-based functional connectivity analysis revealed a DMN covering brain regions commonly associated with this network in the literature (Andrews-Hanna et al., 2014) (Fig. 3*a*). In the upper 50th percentile of this group of unrelated participants, who scored relatively high on ADHD symptoms, the same brain connection identified in our main experiment showed a significant correlation with DSM-oriented AD/H scores [*partial r*_s_ = 0.341, 95% CI (0.07–0.565), *p* = 0.019], corrected for age, gender and mean framewise displacement (Fig. 3*b*). In other words, reduced anti-correlation between the PCC/PCUN and right MFG was associated with greater ADHD symptoms. Moreover, further analysis using the ASR DSM-oriented AD/H sub-scales indicated that the observed relationship was mainly originating from the inattentiveness scores [*partial r*_s_ = 0.397, 95% CI (0.134–0.608), *p* = 0.006], whereas the hyperactivity scores showed no significant correlation with DMN connectivity [*partial r*_s_ = 0.109, 95% CI (−0.174 to 0.375), *p* = 0.465]. Finally, a meta-analytic inquiry into the right MFG on the Neurosynth database revealed terms strongly associated with working memory, attentional load, task demands and other higher executive functions (Fig. 3*c*).

## Discussion

The primary goal of this investigation was to assess whether the default mode functional interactions at rest would show shared alterations in adults with ADHD and their unaffected first-degree relatives as compared to typically developing controls, thus constituting a brain-based endophenotype of this disorder. Our main results revealed significantly reduced anti-correlation (or negative connectivity) of the chosen PCC/PCUN seed, a main hub of the DMN, with a cluster of brain regions centred on the right MFG extending into the IFG that is commonly associated with cognitive control. Moreover, across the whole cohort of participants, the identified functional connection was linked to ADHD symptom severity with performance in the sustained attention task significantly mediating this relationship. Finally, in an external validation step, we illustrated that the same brain connection identified in our main study also explained dimensional variability in ADHD symptoms in an independent cohort of unrelated healthy young adults, providing evidence for the generalisability of our findings. Taken together, such results collectively highlight the central importance of the identified default mode functional interaction as a familial neurocognitive trait in adults with ADHD, hence meeting the criteria for an endophenotype of this disorder.

Previous studies investigating resting-state brain functional connectivity alterations have highlighted DMN connectivity, specifically that of the PCC/PCUN, as a potential new locus of dysfunction in ADHD (Castellanos et al., 2009; Sonuga-Barke & Castellanos, 2007). For example, in comparison with age and gender-matched healthy volunteers, Castellanos and colleagues reported on significantly reduced anti-correlations between the PCC/PCUN and dorsal anterior cingulate cortex in adults with ADHD (Castellanos et al., 2008), which was further replicated in a group of male adolescents (Sun et al., 2012). Though generally in line, our results instead revealed an impairment in the PCC/PCUN interaction with a cluster centred on the right MFG that is typically associated with a large-scale cognitive control system (Dosenbach, Fair, Cohen, Schlaggar, & Petersen, 2008). This is further evidenced by this cluster's strong association with terms linked to higher cognitive processes in our meta-analytic decoding. Importantly, such connectivity alterations mirrored the observed group differences in performance during a sustained attention task, but also showed a stark contrast to the overt clinical phenotype. This ‘exophenotype’ remained in line with the existing diagnosis, revealing heightened symptoms in ADHD probands, which did not reach a diagnostic threshold in either the unaffected first-degree relatives or the typically developing controls, yet it still showed significant links with the identified neurocognitive endophenotypes.

Notably, the PCC/PCUN to MFG connectivity at rest showed a positive correlation with the current total symptoms and a negative correlation with sustained attention task performance, suggesting a close link between the DMN's functional interactions at rest and both inattention and overall symptomatology, which we replicated in an independent cohort of healthy young adults. Collectively, these functional and behavioural links emphasise the significance of the identified disconnection between the PCC/PCUN and right MFG as a neurocognitive trait, and thus a potential endophenotype of ADHD. Moreover, further mediation analysis revealed a significant indirect effect of DMN connectivity on ADHD symptom severity through the participants' performance in the sustained attention task. In a watershed model of biological pathways that link upstream genes to downstream observable phenotypes, Cannon and Keller have argued for the potential utility of identifying intermediary endophenotypes such as neural circuits and more broader cognitive processes in order to improve our understanding of the mechanisms behind complex mental health disorders (Cannon & Keller, 2006). In light of this analogy, our results indicate that alterations in the connectivity of the main DMN hub, namely the PCC/PCUN, may represent an upstream biological dysregulation. This aberrant connectivity in turn may lead to and be further influenced by downstream cognitive dysfunctions such as attentional problems that result in heightened ADHD symptoms. However, taken together with prior reports (Posner et al., 2014), it is clear that further research will be required to decipher the exact neural mechanisms behind the DMN functional interactions that are impaired in ADHD, specifically the relevance of anti-correlations between default mode and frontal brain regions to the neuropathophysiology of this disorder.

Since the initial report by Fox et al., (2005), anti-correlation (or negative connectivity) in the spontaneous BOLD signal fluctuations in rs-fMRI data has been an important topic of debate in the neuroimaging literature. Specifically, the biological basis of this relationship remains largely unknown, with some reports advocating for its foundation in the employed computational techniques (Chai et al., 2012; Murphy et al., 2009). Nevertheless, emerging evidence now highlights the potential contribution of this functional interaction to healthy and adaptive brain processing (Fox, Zhang, Snyder, & Raichle, 2009; Uddin, Kelly, Biswal, Castellanos, & Milham, 2009), with important modifications observed across ageing (Spreng, Stevens, Viviano, & Schacter, 2016) and in mental health disorders (Whitfield-Gabrieli et al., 2009). In parallel, the observed modifications to the segregation between default mode and cognitive control networks have been suggested as a potential mechanism behind the attentional lapses in ADHD (Mills et al., 2018; Posner et al., 2014). However, direct behavioural and functional evidence will be required to not only decipher the biological basis and cognitive relevance of default mode anti-correlations, but also their contribution to the attentional deficits in this disorder.

Originally described as a network of brain regions that showed task-induced deactivations during goal-oriented, attention-demanding paradigms (Mazoyer et al., 2001; Raichle et al., 2001; Shulman et al., 1997) and increased activity in autobiographical memory-based tasks (Addis, Wong, & Schacter, 2007), the role of the DMN in human cognition has long been confined to self-referential internal mentation processes such as mind-wandering or daydreaming (Andrews-Hanna et al., 2014). Combined with evidence relating lapses in attention to DMN activity (Weissman, Roberts, Visscher, & Woldorff, 2006), and alterations in the core DMN regions in ADHD (Castellanos et al., 2008), the overarching hypothesis for the contribution of this large-scale network to ADHD symptomatology has been the excessive generation of mental content that potentially leads to the observed impairments in sustained attention (Posner et al., 2014).

On the other hand, our group has recently shown DMN engagement during the stable application of learned rules in an externally-oriented, attention-demanding cognitive flexibility task (Vatansever, Menon, & Stamatakis, 2017), which highlights the vital role of this network in core cognitive processes that include, yet transcend beyond internal mentation (Vatansever, Manktelow, Sahakian, Menon, & Stamatakis, 2018). Called the ‘autopilot hypothesis’, the potential role that the DMN might play in the maintenance of contextual-goals for fast and efficient processing while avoiding distractors, even that of our thoughts (Bozhilova et al., 2018), might provide a fresh perspective on the DMN's dysfunction in ADHD. In fact, along these lines, we recently provided evidence for the link between impaired context-regulation of thoughts, brain connectivity and ADHD symptom severity (Vatansever, Bozhilova, Asherson, & Smallwood, 2018), highlighting the heterogeneity of this association. In sum, resting-state aberrations found in this study might represent altered DMN contribution to core cognitive and attentional processes.

In relation to the findings of this study, a few limitations should be highlighted. First of all, the results of our study are based on a single brain region within a larger default mode system. Although we used an objective approach to define this seed and focused on a DMN hub that was previously implicated in ADHD, in light of recent evidence indicating functional sub-specialisation within this network (Kernbach et al., 2018), there might be broader ADHD related alterations in the functional interaction of the DMN that should be further considered. For that purpose, other analysis techniques such as independent component analysis (Beckmann, Mackay, Filippini, & Smith, 2009) or the assessment of multivariate interactions of the DMN within the wider complex brain network architecture via graph theoretical analyses might prove important avenues for future research (Fornito & Bullmore, 2015). In addition, our study focused on resting-state alterations in brain connectivity that was related to cognitive performance outside the scanner with a computerised task. Given recent evidence that suggests task-based modifications to the interaction of large-scale systems (Vatansever, Menon, Manktelow, Sahakian, & Stamatakis, 2015), it will be pertinent to directly assess the contribution of default mode interactions to attentional task performance in ADHD. Finally, it is important to note that although all of our analyses were corrected for factors such as age and gender, prior studies do indicate certain characteristic differences related to the subtypes and comorbidities in children with ADHD, that are suggested to normalise in adults (Rucklidge, 2010; Williamson & Johnston, 2015). Hence, future research should explicitly test the potential age and gender effects on the brain-based endophenotypes of ADHD. Notwithstanding, the results of our study highlight shared alterations in resting-state DMN connectivity between ADHD probands and their unaffected first-degree relatives that might have a role into modulating both overall symptoms and inattention.

Overall, this study suggests that the abnormal DMN connectivity is a significant aspect of the adult ADHD neuropathophysiology that could be employed as a latent neurocognitive trait to enhance statistical power of genetic studies in adult ADHD and as a surrogate efficacy endpoint in the research and development of novel pharmaceuticals for treatment. In addition to previous research showing volumetric differences in cortical grey matter and aberrant white matter connections in ADHD (Pironti et al., 2014) the identification of candidate genes linked to neuronal growth, myelination, maintenance and protection (Franke et al., 2012) has strengthened the hypothesis that the observed deficits in ADHD may arise from developmental abnormalities in cortical maturation and pruning that persist into adulthood (Shaw et al., 2007). Such developmental differences may underlie the sustained attention deficits observed in adults with ADHD (Martinussen, Hayden, Hogg-Johnson, & Tannock, 2005), which seem to benefit from methylphenidate administration (Turner et al., 2005) that also normalises DMN alterations (Silk, Malpas, Vance, & Bellgrove, 2016). However, more extensive investigations linking physiological and behavioural endophenotypes with susceptibility genes will be required to find causal interactions. To this end, further research aiming to integrate large-scale datasets (Milham, Fair, Mennes, & Mostofsky, 2012) will be necessary to further characterise the potential endophenotypes of ADHD.

## Supplementary material

For supplementary material accompanying this paper visit https://doi.org/10.1017/S0033291719003374. click here to view supplementary material

## References

[ref1] Achenbach T. M., & Rescorla L. (2003). Manual for the ASEBA adult forms & profiles : for ages 18–59 : adult self-report, adult behavior checklist. Burlington, VT: ASEBA.

[ref2] Addis D. R., Wong A. T., & Schacter D. L. (2007). Remembering the past and imagining the future: common and distinct neural substrates during event construction and elaboration. Neuropsychologia, 45(7), 1363–1377. doi:10.1016/j.neuropsychologia.2006.10.016. 17126370 PMC1894691

[ref3] Andrews-Hanna J. R., Smallwood J., & Spreng R. N. (2014). The default network and self-generated thought: component processes, dynamic control, and clinical relevance. Annals of the New York Academy of Sciences, 1316, 29–52. doi:10.1111/nyas.12360. 24502540 PMC4039623

[ref4] Ashburner J., & Friston K. J. (2005). Unified segmentation. NeuroImage, 26(3), 839–851. doi:10.1016/j.neuroimage.2005.02.018. 15955494

[ref5] Barkley R. A. (2011). Barkley Adult ADHD Rating Scale-IV *(*BAARS-IV*)*. New York: Guilford Press.

[ref6] Barkley R. A., & Fischer M. (2010). The unique contribution of emotional impulsiveness to impairment in major life activities in hyperactive children as adults. Journal of the American Academy of Child & Adolescent Psychiatry, 49(5), 503–513. Retrieved from https://www.ncbi.nlm.nih.gov/pubmed/20431470. 20431470 10.1097/00004583-201005000-00011

[ref7] Barkley R. A., & Murphy K. R. (2006). Attention-deficit hyperactivity disorder: a clinical workbook (3rd ed.). New York: Guilford Press.

[ref8] Beckmann C. F., Mackay C. E., Filippini N., & Smith S. M. (2009). Group comparison of resting-state fMRI data using multi-subject ICA and dual regression. NeuroImage, 47, S148. doi:10.1016/s1053-8119(09)71511-3.

[ref9] Behzadi Y., Restom K., Liau J., & Liu T. T. (2007). A component based noise correction method (CompCor) for BOLD and perfusion based fMRI. NeuroImage, 37(1), 90–101. doi:10.1016/j.neuroimage.2007.04.042. 17560126 PMC2214855

[ref10] Biswal B., Yetkin F. Z., Haughton V. M., & Hyde J. S. (1995). Functional connectivity in the motor cortex of resting human brain using echo-planar MRI. Magnetic Resonance in Medicine, 34, 537–541. Retrieved from http://www.ncbi.nlm.nih.gov/pubmed/8524021. 8524021 10.1002/mrm.1910340409

[ref11] Bozhilova N. S., Michelini G., Kuntsi J., & Asherson P. (2018). Mind wandering perspective on attention-deficit/hyperactivity disorder. Neuroscience and Biobehavioral Reviews, 92, 464–476. 30036553 10.1016/j.neubiorev.2018.07.010 PMC6525148

[ref12] Bright P., Jaldow E., & Kopelman M. D. (2002). The National Adult Reading Test as a measure of premorbid intelligence: a comparison with estimates derived from demographic variables. Journal of the International Neuropsychological Society, 8(6), 847–854. Retrieved from http://www.ncbi.nlm.nih.gov/pubmed/12240749. 12240749 10.1017/s1355617702860131

[ref13] Cannon T. D., & Keller M. C. (2006). Endophenotypes in the genetic analyses of mental disorders. Annual Review of Clinical Psychology, 2, 267–290. doi:10.1146/annurev.clinpsy.2.022305.095232. 17716071

[ref14] Carmona S., Vilarroya O., Bielsa A., Tremols V., Soliva J. C., Rovira M., … Bulbena A. (2005). Global and regional gray matter reductions in ADHD: a voxel-based morphometric study. Neuroscience Letters, 389(2), 88–93. doi:10.1016/j.neulet.2005.07.020. 16129560

[ref15] Castellanos F. X., Kelly C., & Milham M. P. (2009). The restless brain: attention-deficit hyperactivity disorder, resting-state functional connectivity, and intrasubject variability. Canadian Journal of Psychiatry, 54(10), 665–672. doi:10.1177/070674370905401003. 19835673 PMC3876940

[ref16] Castellanos F. X., Margulies D. S., Kelly C., Uddin L. Q., Ghaffari M., Kirsch A., … Milham M. P. (2008). Cingulate-precuneus interactions: a new locus of dysfunction in adult attention-deficit/hyperactivity disorder. Biological Psychiatry, 63(3), 332–337. doi:10.1016/j.biopsych.2007.06.025. 17888409 PMC2745053

[ref17] Castellanos F. X., & Tannock R. (2002). Neuroscience of attention-deficit/hyperactivity disorder: the search for endophenotypes. Nature Reviews Neuroscience, 3(8), 617–628. doi:10.1038/nrn896. 12154363

[ref18] Chabernaud C., Mennes M., Kelly C., Nooner K., Di Martino A., Castellanos F. X., & Milham M. P. (2012). Dimensional brain-behavior relationships in children with attention-deficit/hyperactivity disorder. Biological Psychiatry, 71(5), 434–442. doi:10.1016/j.biopsych.2011.08.013. 21974788 PMC3568534

[ref19] Chai X. J., Castanon A. N., Ongur D., & Whitfield-Gabrieli S. (2012). Anticorrelations in resting state networks without global signal regression. NeuroImage, 59(2), 1420–1428. doi:10.1016/j.neuroimage.2011.08.048. 21889994 PMC3230748

[ref20] Chen Q., Brikell I., Lichtenstein P., Serlachius E., Kuja-Halkola R., Sandin S., & Larsson H. (2017). Familial aggregation of attention-deficit/hyperactivity disorder. Journal of Child Psychology and Psychiatry, and Allied Disciplines, 58(3), 231–239. doi:10.1111/jcpp.12616. 27545745

[ref21] Dosenbach N. U., Fair D. A., Cohen A. L., Schlaggar B. L., & Petersen S. E. (2008). A dual-networks architecture of top-down control. Trends in Cognitive Sciences, 12(3), 99–105. doi:10.1016/j.tics.2008.01.001. 18262825 PMC3632449

[ref22] Doyle A. E., Willcutt E. G., Seidman L. J., Biederman J., Chouinard V. A., Silva J., & Faraone S. V. (2005). Attention-deficit/hyperactivity disorder endophenotypes. Biological Psychiatry, 57(11), 1324–1335. doi:S0006-3223(05)00355-0 [pii]. 10.1016/j.biopsych.2005.03.015 [doi]. 15950005

[ref23] Elton A., Alcauter S., & Gao W. (2014). Network connectivity abnormality profile supports a categorical-dimensional hybrid model of ADHD. Human Brain Mapping, 35(9), 4531–4543. doi:10.1002/hbm.22492. 24615988 PMC4213949

[ref24] Fair D. A., Schlaggar B. L., Cohen A. L., Miezin F. M., Dosenbach N. U., Wenger K. K., … Petersen S. E. (2007). A method for using blocked and event-related fMRI data to study “resting state” functional connectivity. NeuroImage, 35(1), 396–405. doi:10.1016/j.neuroimage.2006.11.051. 17239622 PMC2563954

[ref25] Faraone S. V. (2007). ADHD in adults – a familiar disease with unfamiliar challenges. CNS Spectrums, 12(12 Suppl 23), 14–17. Retrieved from https://www.ncbi.nlm.nih.gov/pubmed/18389930. 10.1017/s1092852900003801 18389930

[ref26] Faraone S. V., & Larsson H. (2019). Genetics of attention deficit hyperactivity disorder. Molecular Psychiatry, 24(4), 562–575. doi:10.1038/s41380-018-0070-0. 29892054 PMC6477889

[ref27] Finn E. S., Shen X., Scheinost D., Rosenberg M. D., Huang J., Chun M. M., … Constable R. T. (2015). Functional connectome fingerprinting: identifying individuals using patterns of brain connectivity. Nature Neuroscience, 18(11), 1664–1671. doi:10.1038/nn.4135. 26457551 PMC5008686

[ref28] Fornito A., & Bullmore E. T. (2015). Connectomics: a new paradigm for understanding brain disease. European Neuropsychopharmacology, 25(5), 733–748. doi:10.1016/j.euroneuro.2014.02.011. 24726580

[ref29] Fox M. D., & Greicius M. (2010). Clinical applications of resting state functional connectivity. Frontiers in Systems Neuroscience, 4, 19. doi:10.3389/fnsys.2010.00019. 20592951 PMC2893721

[ref30] Fox M. D., Snyder A. Z., Vincent J. L., Corbetta M., Van Essen D. C., & Raichle M. E. (2005). The human brain is intrinsically organized into dynamic, anticorrelated functional networks. Proceedings of the National Academy of Sciences of the United States of America, 102(27), 9673–9678. doi:10.1073/pnas.0504136102. 15976020 PMC1157105

[ref31] Fox M. D., Zhang D., Snyder A. Z., & Raichle M. E. (2009). The global signal and observed anticorrelated resting state brain networks. Journal of Neurophysiology, 101(6), 3270–3283. doi:10.1152/jn.90777.2008. 19339462 PMC2694109

[ref32] Franke B., Faraone S. V., Asherson P., Buitelaar J., Bau C. H., Ramos-Quiroga J. A., … International Multicentre persistent, A. C. (2012). The genetics of attention deficit/hyperactivity disorder in adults, a review. Molecular Psychiatry, 17(10), 960–987. doi:10.1038/mp.2011.138. 22105624 PMC3449233

[ref33] Fransson P., & Marrelec G. (2008). The precuneus/posterior cingulate cortex plays a pivotal role in the default mode network: evidence from a partial correlation network analysis. NeuroImage, 42(3), 1178–1184. doi:10.1016/j.neuroimage.2008.05.059. 18598773

[ref34] Friston K. J. (1994). Functional and effective connectivity in neuroimaging: a synthesis. Human Brain Mapping, 2(1–2), 56–78. doi:10.1002/hbm.460020107.

[ref35] Gau S. S., & Huang W. L. (2014). Rapid visual information processing as a cognitive endophenotype of attention deficit hyperactivity disorder. Psychological Medicine, 44(2), 435–446. doi:10.1017/S0033291713000640. 23561037

[ref36] Glahn D. C., Winkler A. M., Kochunov P., Almasy L., Duggirala R., Carless M. A., … Blangero J. (2010). Genetic control over the resting brain. Proceedings of the National Academy of Sciences of the United States of America, 107(3), 1223–1228. doi:10.1073/pnas.0909969107. 20133824 PMC2824276

[ref37] Glasser M. F., Sotiropoulos S. N., Wilson J. A., Coalson T. S., Fischl B., Andersson J. L., … Consortium W. U.-M. H. (2013). The minimal preprocessing pipelines for the Human Connectome Project. NeuroImage, 80, 105–124. doi:10.1016/j.neuroimage.2013.04.127. 23668970 PMC3720813

[ref38] Gottesman I. I., & Gould T. D. (2003). The endophenotype concept in psychiatry: etymology and strategic intentions. American Journal of Psychiatry, 160(4), 636–645. 10.1176/appi.ajp.160.4.636 12668349

[ref39] Gualtieri C. T., Wargin W., Kanoy R., Patrick K., Shen C. D., Youngblood W., … Breese G. R. (1982). Clinical studies of methylphenidate serum levels in children and adults. Journal of the American Academy of Child Psychiatry, 21(1), 19–26. Retrieved from https://www.ncbi.nlm.nih.gov/pubmed/7096827. 7096827 10.1097/00004583-198201000-00005

[ref40] Hayes A. F. (2018). Introduction to mediation, moderation, and conditional process analysis: a regression-based approach (2nd ed.). New York: Guilford Press.

[ref41] Hinney A., Scherag A., Jarick I., Albayrak O., Putter C., Pechlivanis S., … Psychiatric G. C. A. S. (2011). Genome-wide association study in German patients with attention deficit/hyperactivity disorder. American Journal of Medical Genetics. Part B, Neuropsychiatric Genetics, 156B(8), 888–897. doi:10.1002/ajmg.b.31246. 22012869

[ref42] Kernbach J. M., Yeo B. T. T., Smallwood J., Margulies D. S., Thiebaut de Schotten M., Walter H., … Bzdok D. (2018). Subspecialization within default mode nodes characterized in 10,000 UK Biobank participants. Proceedings of the National Academy of Sciences of the United States of America, 115(48), 12295–12300. doi:10.1073/pnas.1804876115. 30420501 PMC6275484

[ref43] Kieling C., Kieling R. R., Rohde L. A., Frick P. J., Moffitt T., Nigg J. T., … Castellanos F. X. (2010). The age at onset of attention deficit hyperactivity disorder. American Journal of Psychiatry, 167(1), 14–16. doi:10.1176/appi.ajp.2009.09060796. PMC4478075 20068122

[ref44] Konrad K., & Eickhoff S. B. (2010). Is the ADHD brain wired differently? A review on structural and functional connectivity in attention deficit hyperactivity disorder. Human Brain Mapping, 31(6), 904–916. doi:10.1002/hbm.21058. 20496381 PMC6871159

[ref45] Korgaonkar M. S., Ram K., Williams L. M., Gatt J. M., & Grieve S. M. (2014). Establishing the resting state default mode network derived from functional magnetic resonance imaging tasks as an endophenotype: a twins study. Human Brain Mapping, 35(8), 3893–3902. doi:10.1002/hbm.22446. 24453120 PMC6869646

[ref46] Leech R., & Sharp D. J. (2014). The role of the posterior cingulate cortex in cognition and disease. Brain, 137(Pt 1), 12–32. doi:10.1093/brain/awt162. 23869106 PMC3891440

[ref47] Maldjian J. A., Laurienti P. J., Kraft R. A., & Burdette J. H. (2003). An automated method for neuroanatomic and cytoarchitectonic atlas-based interrogation of fMRI data sets. NeuroImage, 19(3), 1233–1239. Retrieved from http://www.ncbi.nlm.nih.gov/pubmed/12880848. 12880848 10.1016/s1053-8119(03)00169-1

[ref48] Martinussen R., Hayden J., Hogg-Johnson S., & Tannock R. (2005). A meta-analysis of working memory impairments in children with attention-deficit/hyperactivity disorder. Journal of the American Academy of Child & Adolescent Psychiatry, 44(4), 377–384. doi:10.1097/01.chi.0000153228.72591.73. 15782085

[ref49] Mazoyer B., Zago L., Mellet E., Bricogne S., Etard O., Houdé O., … Tzourio-Mazoyer N. (2001). Cortical networks for working memory and executive functions sustain the conscious resting state in man. Brain Research Bulletin, 54(3), 287–298. Retrieved from http://www.ncbi.nlm.nih.gov/pubmed/11287133. 11287133 10.1016/s0361-9230(00)00437-8

[ref50] McLean A., Dowson J., Toone B., Young S., Bazanis E., Robbins T. W., & Sahakian B. J. (2004). Characteristic neurocognitive profile associated with adult attention-deficit/hyperactivity disorder. Psychological Medicine, 34(4), 681–692. doi:10.1017/S0033291703001296. 15099422

[ref51] Mick E., & Faraone S. V. (2008). Genetics of attention deficit hyperactivity disorder. Child and Adolescent Psychiatric Clinics of North America, 17(2), 261–284, vii–viii. doi:10.1016/j.chc.2007.11.011. 18295146

[ref52] Milham M. P., Fair D., Mennes M., & Mostofsky S. H. (2012). The ADHD-200 Consortium: a model to advance the translational potential of neuroimaging in clinical neuroscience. Frontiers in Systems Neuroscience, 6, 62. doi:10.3389/fnsys.2012.00062. 22973200 PMC3433679

[ref53] Mills B. D., Miranda-Dominguez O., Mills K. L., Earl E., Cordova M., Painter J., … Fair D. A. (2018). ADHD and attentional control: impaired segregation of task positive and task negative brain networks. Network Neuroscience, 2(2), 200–217. doi:10.1162/netn_a_00034. 30215033 PMC6130439

[ref54] Murphy K., Birn R. M., Handwerker D. A., Jones T. B., & Bandettini P. A. (2009). The impact of global signal regression on resting state correlations: are anti-correlated networks introduced? NeuroImage, 44(3), 893–905. doi:10.1016/j.neuroimage.2008.09.036. 18976716 PMC2750906

[ref55] Pironti V. A., Lai M. C., Muller U., Dodds C. M., Suckling J., Bullmore E. T., & Sahakian B. J. (2014). Neuroanatomical abnormalities and cognitive impairments are shared by adults with attention-deficit/hyperactivity disorder and their unaffected first-degree relatives. Biological Psychiatry, 76(8), 639–647. doi:10.1016/j.biopsych.2013.09.025. 24199662 PMC4183379

[ref56] Posner J., Park C., & Wang Z. (2014). Connecting the dots: a review of resting connectivity MRI studies in attention-deficit/hyperactivity disorder. Neuropsychology Review, 24(1), 3–15. doi:10.1007/s11065-014-9251-z. 24496902 PMC4119002

[ref57] Raichle M. E., MacLeod A. M., Snyder A. Z., Powers W. J., Gusnard D. A., & Shulman G. L. (2001). A default mode of brain function. Proceedings of the National Academy of Sciences of the United States of America, 98(2), 676–682. doi:10.1073/pnas.98.2.676. 11209064 PMC14647

[ref58] Robbins T. W., James M., Owen A. M., & Sahakian B. J. (1994). Cambridge Neuropsychological Test Automated Battery (CANTAB): a factor analytic study of a large sample of normal elderly volunteers. Dementia (Basel, Switzerland), 5(5), 266–281. Retrieved from http://search.ebscohost.com/login.aspx?direct=true&db=psyh&AN=1995-08060-001&site=ehost-live&scope=site. 10.1159/000106735 7951684

[ref59] Rubin T. N., Koyejo O., Gorgolewski K. J., Jones M. N., Poldrack R. A., & Yarkoni T. (2017). Decoding brain activity using a large-scale probabilistic functional-anatomical atlas of human cognition. PLoS Computational Biology, 13(10), e1005649. doi:10.1371/journal.pcbi.1005649. 29059185 PMC5683652

[ref60] Rucklidge J. J. (2010). Gender differences in attention-deficit/hyperactivity disorder. The Psychiatric Clinics of North America, 33(2), 357–373. doi:10.1016/j.psc.2010.01.006. 20385342

[ref61] Sahakian B. J., & Owen A. M. (1992). Computerized assessment in neuropsychiatry using CANTAB: discussion paper. Journal of the Royal Society of Medicine, 85(7), 399–402. Retrieved from https://www.ncbi.nlm.nih.gov/pubmed/1629849. 1629849 PMC1293547

[ref62] Shaw P., Eckstrand K., Sharp W., Blumenthal J., Lerch J. P., Greenstein D., … Rapoport J. L. (2007). Attention-deficit/hyperactivity disorder is characterized by a delay in cortical maturation. Proceedings of the National Academy of Sciences of the United States of America, 104(49), 19649–19654. doi:10.1073/pnas.0707741104. 18024590 PMC2148343

[ref63] Shaw P., Lerch J., Greenstein D., Sharp W., Clasen L., Evans A., … Rapoport J. (2006). Longitudinal mapping of cortical thickness and clinical outcome in children and adolescents with attention-deficit/hyperactivity disorder. Archives of General Psychiatry, 63(5), 540–549. doi:10.1001/archpsyc.63.5.540. 16651511

[ref64] Sheehan D. V., Lecrubier Y., Sheehan K. H., Amorim P., Janavs J., Weiller E., … Dunbar G. C. (1998). The Mini-international neuropsychiatric interview (M.I.N.I.): the development and validation of a structured diagnostic psychiatric interview for DSM-IV and ICD-10. Journal of Clinical Psychiatry, 59(Suppl 20), 22–33; quiz 34–57. Retrieved from https://www.ncbi.nlm.nih.gov/pubmed/9881538. 9881538

[ref65] Shrout P. E., & Bolger N. (2002). Mediation in experimental and nonexperimental studies: new procedures and recommendations. Psychological Methods, 7(4), 422–445. Retrieved from https://www.ncbi.nlm.nih.gov/pubmed/12530702. 12530702

[ref66] Shulman G. L., Fiez J. A., Corbetta M., Buckner R. L., Miezin F. M., Raichle M. E., & Petersen S. E. (1997). Common blood flow changes across visual tasks: II. Decreases in cerebral cortex. Journal of Cognitive Neuroscience, 9(5), 648–663. doi:10.1162/jocn.1997.9.5.648. 23965122

[ref67] Silk T. J., Malpas C., Vance A., & Bellgrove M. A. (2016). The effect of single-dose methylphenidate on resting-state network functional connectivity in ADHD. Brain Imaging and Behavior, 11(5), 1422–1431. doi:10.1007/s11682-016-9620-8. 27734305

[ref68] Smith S. M., Miller K. L., Salimi-Khorshidi G., Webster M., Beckmann C. F., Nichols T. E., … Woolrich M. W. (2011). Network modelling methods for fMRI. NeuroImage, 54(2), 875–891. doi:10.1016/j.neuroimage.2010.08.063. 20817103

[ref69] Sonuga-Barke E. J. S., & Castellanos F. X. (2007). Spontaneous attentional fluctuations in impaired states and pathological conditions: a neurobiological hypothesis. Neuroscience and Biobehavioral Reviews, 31, 977–986. doi:10.1016/j.neubiorev.2007.02.005. 17445893

[ref70] Spreng R. N., Stevens W. D., Viviano J. D., & Schacter D. L. (2016). Attenuated anticorrelation between the default and dorsal attention networks with aging: evidence from task and rest. Neurobiology of Aging, 45, 149–160. doi:10.1016/j.neurobiolaging.2016.05.020. 27459935 PMC5003045

[ref71] Sun L., Cao Q., Long X., Sui M., Cao X., Zhu C., … Wang Y. (2012). Abnormal functional connectivity between the anterior cingulate and the default mode network in drug-naive boys with attention deficit hyperactivity disorder. Psychiatry Research, 201(2), 120–127. doi:10.1016/j.pscychresns.2011.07.001. 22424873

[ref72] Thapar A., O'Donovan M., & Owen M. J. (2005). The genetics of attention deficit hyperactivity disorder. Human Molecular Genetics, 14 Spec No. 2, R275–R282. doi:10.1093/hmg/ddi263. 16244326

[ref73] Turner D. C., Blackwell A. D., Dowson J. H., McLean A., & Sahakian B. J. (2005). Neurocognitive effects of methylphenidate in adult attention-deficit/hyperactivity disorder. Psychopharmacology *(*Berlin*)*, 178(2–3), 286–295. doi:10.1007/s00213-004-1993-5. 15338103

[ref74] Uddin L. Q., Kelly A. M., Biswal B. B., Castellanos F. X., & Milham M. P. (2009). Functional connectivity of default mode network components: correlation, anticorrelation, and causality. Human Brain Mapping, 30(2), 625–637. doi:10.1002/hbm.20531. 18219617 PMC3654104

[ref75] Valera E. M., Faraone S. V., Murray K. E., & Seidman L. J. (2007). Meta-analysis of structural imaging findings in attention-deficit/hyperactivity disorder. Biological Psychiatry, 61(12), 1361–1369. doi:10.1016/j.biopsych.2006.06.011. 16950217

[ref76] Van Essen D. C., Smith S. M., Barch D. M., Behrens T. E., Yacoub E., Ugurbil K., & Consortium W. U.-M. H. (2013). The WU-Minn Human Connectome Project: an overview. NeuroImage, 80, 62–79. doi:10.1016/j.neuroimage.2013.05.041. 23684880 PMC3724347

[ref77] Vatansever D., Bozhilova N. S., Asherson P., & Smallwood J. (2018). The devil is in the detail: exploring the intrinsic neural mechanisms that link attention-deficit/hyperactivity disorder symptomatology to ongoing cognition. Psychological Medicine, 49(7), 1185–1194. doi:10.1017/S0033291718003598. 30514410

[ref78] Vatansever D., Manktelow A. E., Sahakian B. J., Menon D. K., & Stamatakis E. A. (2016). Cognitive flexibility: a default network and basal ganglia connectivity perspective. Brain Connectivity, 6(3), 201–207. doi:10.1089/brain.2015.0388. 26652748 PMC5118962

[ref79] Vatansever D., Manktelow A. E., Sahakian B. J., Menon D. K., & Stamatakis E. A. (2018). Default mode network engagement beyond self-referential internal mentation. Brain Connectivity. doi:10.1089/brain.2017.0489. 29366339

[ref80] Vatansever D., Menon D. K., Manktelow A. E., Sahakian B. J., & Stamatakis E. A. (2015). Default mode dynamics for global functional integration. Journal of Neuroscience, 35(46), 15254–15262. doi:10.1523/JNEUROSCI.2135-15.2015. 26586814 PMC4649001

[ref81] Vatansever D., Menon D. K., & Stamatakis E. A. (2017). Default mode contributions to automated information processing. Proceedings of the National Academy of Sciences of the United States of America, 114(48), 12821–12826. doi:10.1073/pnas.1710521114. 29078345 PMC5715758

[ref82] Waskom M., Botvinnik O., O'Kane D., Hobson P., Ostblom J., Lukauskas S., … Qalieh A. (2018). mwaskom/seaborn: v0.9.0 (July 2018). doi:10.5281/ZENODO.1313201.

[ref83] Weissman D. H., Roberts K. C., Visscher K. M., & Woldorff M. G. (2006). The neural bases of momentary lapses in attention. Nature Neuroscience, 9(7), 971–978. doi:10.1038/nn1727. 16767087

[ref84] Whitfield-Gabrieli S., & Nieto-Castanon A. (2012). Conn: a functional connectivity toolbox for correlated and anticorrelated brain networks. Brain Connectivity, 2(3), 125–141. doi:10.1089/brain.2012.0073. 22642651

[ref85] Whitfield-Gabrieli S., Thermenos H. W., Milanovic S., Tsuang M. T., Faraone S. V., McCarley R. W., & Seidman L. J. (2009). Hyperactivity and hyperconnectivity of the default network in schizophrenia and in first-degree relatives of persons with schizophrenia. Proceedings of the National Academy of Sciences of the United States of America, 106(4), 1279–1284. doi:10.1073/pnas.0809141106. 19164577 PMC2633557

[ref86] Williamson D., & Johnston C. (2015). Gender differences in adults with attention-deficit/hyperactivity disorder: a narrative review. Clinical Psychology Review, 40, 15–27. doi:10.1016/j.cpr.2015.05.005. 26046624

[ref87] Yarkoni T., Poldrack R. A., Nichols T. E., Van Essen D. C., & Wager T. D. (2011). Large-scale automated synthesis of human functional neuroimaging data. Nature Methods, 8(8), 665–670. doi:10.1038/nmeth.1635. 21706013 PMC3146590

