# Supplementary material for: Shared alterations in resting-state brain connectivity in adults with attention-deficit/hyperactivity disorder and their unaffected first-degree relatives
Source: Psychol Med. 2019 Nov 26;51(2):329–39. doi: 10.1017/S0033291719003374 (PMC7893505; doi:10.1017/S0033291719003374)
Supplement: Supplementary file 1 [file S0033291719003374sup001.docx]

**Supplementary Materials: Shared alterations in resting state brain connectivity in adults with attention-deficit/hyperactivity disorder and their unaffected first-degree relatives**

**Authors:** Valentino Antonio Pironti^1,2,6^*, Deniz Vatansever^1,3,4,5^*, Barbara Jacquelyn Sahakian^1,2,3^

**Affiliations:**

^1^Department of Psychiatry, School of Clinical Medicine, University of Cambridge, Cambridge, UK

^2^Behavioural and Clinical Neuroscience Institute, University of Cambridge, Cambridge, UK

^3^Institute of Science and Technology for Brain-inspired Intelligence, Fudan University, Shanghai, PR China

^4^Division of Anaesthesia, School of Clinical Medicine, University of Cambridge, Cambridge, UK

^5^Department of Psychology, University of York, Heslington, York, UK

^6^Suno Innova Ltd, Unit 6, 109 Cambridge Road Industrial Estate, Cambridge, UK

*** These authors contributed equally to this work.**

**Address for correspondence:**

Deniz Vatansever, PhD

Institute of Science and Technology for Brain-inspired Intelligence,

Fudan University

Shanghai, PR China 200433

Email: deniz@fudan.edu.cn

Table of Contents

[Supplementary Results 2](#_Toc19027151)

[Demographic Assessment 2](#_Toc19027152)

[Clinical Assessment 2](#_Toc19027153)

[Association of Brain Connectivity, Cognition and ADHD Symptomatology 3](#_Toc19027154)

[References 6](#_Toc19027155)

Supplementary Results

Demographic Assessment

Multivariate and univariate ANOVAs as well as post-hoc t-tests were used to ensure matching in age, gender and NART full IQ scores across the three groups (i.e. ADHD probands, their first-degree relatives and typically developing controls) and to identify potential confounds that could be used as covariates of no interest in the subsequent analyses. Table S1 summarises the demographic information and the accompanying statistical test results. Multivariate ANOVA Wilk Lambda test indicated a significant difference in the dependent variables across groups (*F_(6,110)_* = 3.137, *p* = 0.007). Univariate ANOVAs across these measures suggested that age and gender were matched between groups, and that the disparity arose mainly from NART full IQ scores (*F_(2,57)_* = 3.679, *p* = 0.031, *partial η^2^* = 0.114). Post-hoc t-tests indicated that the ADHD group performed significantly worse than the typically developing control group (*t_(57)_* = -2.652, 95% CI [-7.429, -1.037], *p* = .010, *partial η^2^* = 0.110). Since NART full IQ scores were significantly correlated with age (r_s_ = 0.363, *p* = 0.004), we also ran an additional univariate ANCOVA correcting for age and gender. The results of this test also displayed a significant difference (*F_(2,55)_* = 4.284, *p* = 0.019, *partial η^2^* = 0.135), specifically between the ADHD and control groups (*t_(55)_* = -2.696, 95% CI [-7.465, -1.099], *p* = .009, *partial η^2^* = 0.117). In order to ensure that we removed the potential effects of this demographic information, age, gender and NART full IQ scores were all used as covariates in the subsequent analyses.

Table S1. Sample characteristics of the main study cohort.

|  | ADHD | | Relatives | | Controls | | ANOVA | |
| --- | --- | --- | --- | --- | --- | --- | --- | --- |
|  | Mean | SD | Mean | SD | Mean | SD | *F_(2,57)_* | *p* |
| Age (years) | 32.20 | 10.31 | 38.85 | 15.31 | 32.55 | 5.8 | 2.245 | 0.115 |
| Gender (% females) | 15 |  | 50 |  | 35 |  | 2.905 | 0.063 |
| NART Full IQ | 115.26 | 6.15 | 116.59 | 5.28 | 119.49 | 3.27 | 3.679 | 0.031 |

Clinical Assessment

In the main study cohort, ADHD symptomatology was assessed using the Barkley Adult ADHD Rating Scale Version IV (BAARS-IV). BAARS-IV is a screening tool that examines symptoms and dysfunctions associated with ADHD, based on the Diagnostic and Statistical Manual of Mental Disorders Version IV (DSM-IV). The scale includes separate forms for both childhood and current symptoms (i.e. within the past 6 months). The symptoms are rated on a 4-point scale across 18 items in which the ratings of “Often [3]” and “Very Often [4]” denote the number of symptoms, while the total scores indicate the total symptom score ratings ([Barkley, 2011](#_ENREF_2)). The self-report BAARS-IV current total symptom scores were employed in this study.

Table S2 summarises the differences in BAARS-IV clinical symptom scores across groups that was significant at the multivariate level using Wilk Lambda test (*F_(4,106)_* = 22.459, *p* < 0.001). Univariate ANCOVAs and post-hoc t-tests revealed that for all measures, the ADHD probands differed significantly from both their first-degree relatives (*p* < 0.001) and healthy controls (*p* < 0.001); however, the latter two groups did not show a significant difference (*p* > 0.05). Given their high correlation with each other, the BAARS-IV current total score was used as the assessment of choice for the subsequent analyses as opposed to the inattentive and hyper-impulsive sub-scales. All ANCOVAs were corrected for age, gender and NART full IQ score and the post-hoc t-tests were multiple comparison corrected using the Bonferroni method.

Table S2. Clinical assessment scores for ADHD symptomatology in the main study cohort.

|  | ADHD | | Relatives | | Controls | | ANOVA | |
| --- | --- | --- | --- | --- | --- | --- | --- | --- |
|  | Mean | SD | Mean | SD | Mean | SD | *F_(2,54)_* | *p* |
| BAARS-IV Current Total Score | 36.15 | 12.39 | 10.00 | 7.38 | 5.20 | 4.29 | 62.328 | <0.001 |
| BAARS-IV Current Inattentive Score | 17.75 | 5.96 | 4.75 | 3.89 | 2.70 | 2.77 | 59.347 | <0.001 |
| BAARS-IV Current Hyperactive/Impulsive Score | 18.40 | 6.98 | 5.25 | 4.13 | 2.50 | 2.50 | 50.200 | <0.001 |

In the Human Connectome Project (HCP) sample, the Achenbach Adult Self-Report (ASR) was used to examine ADHD symptomatology. ASR is a questionnaire that aims to assess aspects of adaptive psychological functioning and mental health problems ([Achenbach and Rescorla, 2003](#_ENREF_1)). A total of 126 items are rated on a 3-point scale in which 13 of the items form part of the DSM-Oriented sub-scale of attention-deficit and hyperactivity (AD/H) problems. The raw ASR total AD/H symptom scores were employed in this study. It is important to note that in order to avoid floor effects in this neurotypical cohort, only the data from participants above the median ASR DSM-Oriented AD/H score were included in the correlation analysis between brain connectivity and ADHD sympto-matology (mean age = 29.11, SD = 3.676, female to male ratio = 54/46).

Association of Brain Connectivity, Cognition and ADHD Symptomatology

The employed brain connectivity, cognition and ADHD symptomatology measurements all showed significant links across the three groups. Participants who scored higher on BAARS-IV current total symptom scores showed reduced DMN anti-correlation and a smaller number of total hits on the RVP sustained attention task, which in turn was linked to reduced DMN anti-correlation. The BAARS-IV current total symptoms and the RVP total hits were not normally distributed (Q-Q Plots and Shapiro-Wilk Test < 0.05), and thus non-parametric partial Spearman’s correlation coefficient (*r_s_*) was reported in the main manuscript, correcting for age, gender, NART full IQ score and mean framewise displace-ment (Table S3). The significance level of the observed correlations survived multiple comparisons using Bonferroni correction. The same analysis was also carried out for the BAARS-IV current inattentive and current hyper-impulsive symptom sub-scales, which revealed comparable results (Tables S4-S5).

Table S3. Non-parametric partial Spearman’s correlations of ADHD symptomatology (BAARS-IV Current Total Symptoms), cognitive assessment and resting state brain connectivity (PCC/PCUN to right MFG), correcting for age, gender, NART full IQ score and mean framewise displacement.

|  |  | BAARS-IV Current Total Symptoms | RVP Total Hits | PCC/PCUN and Right MFG Connectivity |
| --- | --- | --- | --- | --- |
| BAARS-IV Current Total Symptoms | Correlation Coefficient | 1.000 | -0.456^**^  95% CI [-.636, -.229] | 0.405^**^  95% CI [.168, .598] |
|  | Significance (2-tailed) |  | 0.000 | 0.002 |
|  | df |  | 54 | 54 |
| RVP Total Hits | Correlation Coefficient | -0.456^**^  95% CI [-.636, -.229] | 1.000 | -0.360^**^  95% CI [-.563, -.117] |
|  | Significance (2-tailed) | 0.000 |  | 0.006 |
|  | df | 54 |  | 54 |
| PCC/PCUN and Right MFG Connectivity | Correlation Coefficient | 0.405^**^  95% CI [.168, .598] | -0.360^**^  95% CI [-.563, -.117] | 1.000 |
|  | Significance (2-tailed) | 0.002 | 0.006 |  |
|  | df | 54 | 54 |  |
| **. Correlation is significant at the 0.01 level (2-tailed). | | | | |
|  | | | | |

Table S4. Non-parametric partial Spearman’s correlation of ADHD symptomatology (BAARS-IV Current Inattentive Symptoms), cognitive assessment and resting state brain connectivity (PCC/PCUN to right MFG), correcting for age, gender, NART full IQ score and mean framewise displacement.

|  | | BAARS-IV Current Inattentive Symptoms | RVP Total Hits | PCC/PCUN and Right MFG Connectivity |
| --- | --- | --- | --- | --- |
| BAARS-IV Current Inattentive Symptoms | Correlation Coefficient | 1.000 | -0.446^**^  95% CI [-.629, -.217] | 0.401^**^  95% CI [.164, .594] |
|  | Significance (2-tailed) |  | 0.001 | 0.002 |
|  | df |  | 54 | 54 |
| RVP Total Hits | Correlation Coefficient | -0.446^**^  95% CI [-.629, -.217] | 1.000 | -0.360^**^  95% CI [-.563, -.117] |
|  | Significance (2-tailed) | 0.001 |  | 0.006 |
|  | df | 54 |  | 54 |
| PCC/PCUN and Right MFG Connectivity | Correlation Coefficient | 0.401^**^  95% CI [.164, .594] | -0.360^**^  95% CI [-.563, -.117] | 1.000 |
|  | Significance (2-tailed) | 0.002 | 0.006 |  |
|  | df | 54 | 54 |  |
| **. Correlation is significant at the 0.01 level (2-tailed). | | | | |

Table S5. Non-parametric partial Spearman’s correlation of ADHD symptoms (BAARS-IV current Hyper-Impulsive Symptoms), cognitive assessment and resting state brain connectivity (PCC/PCUN to right MFG), correcting for age, gender, NART full IQ score and mean framewise displacement.

|  | | BAARS-IV Current Hyper-Impulsive Symptoms | RVP Total Hits | PCC/PCUN and Right MFG Connectivity |
| --- | --- | --- | --- | --- |
| BAARS-IV Current Hyper-Impulsive Symptoms | Correlation Coefficient | 1.000 | -0.417^**^  95% CI [-.607, -.182] | 0.406^**^  95% CI [.170, .598] |
|  | Significance (2-tailed) |  | 0.001 | 0.002 |
|  | df |  | 54 | 54 |
| RVP Total Hits | Correlation Coefficient | -0.417^**^  95% CI [-.607, -.182] | 1.000 | -0.360^**^  95% CI [-.563, -.117] |
|  | Significance (2-tailed) | 0.001 |  | 0.006 |
|  | df | 54 |  | 54 |
| PCC/PCUN and Right MFG Connectivity | Correlation Coefficient | 0.406^**^  95% CI [.170, .598] | -0.360^**^  95% CI [-.563, -.117] | 1.000 |
|  | Significance (2-tailed) | 0.002 | 0.006 |  |
|  | df | 54 | 54 |  |
| **. Correlation is significant at the 0.01 level (2-tailed). | | | | |

References

**Achenbach, T. M. & Rescorla, L.** (2003). *Manual for the ASEBA adult forms & profiles : for ages 18-59 : adult self-report, adult behavior checklist*. ASEBA: Burlington, VT.

**Barkley, R. A.** (2011). *Barkley Adult ADHD Rating Scale-IV (BAARS-IV)*. Guilford Press: New York.
